# Delirium, dementia, and depression (3D) assessment of older patients in the emergency department: 5-year survival analysis

**DOI:** 10.3906/sag-2110-207

**Published:** 2021-12-16

**Authors:** İsa KILIÇASLAN, Myat Soe THET, Berkay KARAHACIOĞLU, Merve SEVİM, Zekeriya ÜLGER, Mehmet Ali ASLANER, Banu KILIÇASLAN

**Affiliations:** 1 Department of Emergency Medicine, Faculty of Medicine, Gazi University, Ankara, Turkey; 2 School of Medicine, Faculty of Medicine, Gazi University, Ankara, Turkey; 3 Division of Geriatric Medicine, Department of Internal Medicine, Faculty of Medicine, Gazi University, Ankara, Turkey; 4 Department of Anesthesiology and Reanimation, Faculty of Medicine, Hacettepe University, Ankara, Turkey

**Keywords:** Delirium, dementia, depression, survival, older mental health disorder, geriatric emergency department

## Abstract

**Background:**

While physicians tend to emphasize on physical medical problems, behavioral and cognitive disorders of geriatric patients are usually missed, especially in emergency settings. The aim of the study was to determine the prevalence of delirium, dementia, and depression (3D) among older patients (≥65 years old) in the Emergency Department (ED) and to evaluate the effect of geriatric 3D on the 6-month and 5-year mortality.

**Materials and methods:**

This was a prospective, observational cohort study, including 415 patients from eligible 512 consecutive older patients, who are 65 years of age or older, presenting to the ED of a tertiary care university hospital. Geriatric delirium, dementia, and depression were prospectively evaluated using Confusion Assessment Method, Quick Confusion Scale, and Geriatric Depression Scale-15, respectively. Premorbid functional status was determined by Barthel Index. The Charlson Comorbidity Index was used to measure the comorbid burden. After enrollment, patients were screened for 6-month and 5-year survival rates via the Government Death Reporting System records. The Kaplan–Meier method and Cox proportional hazards analysis was used for survival analysis.

**Results:**

Among the study population, the prevalence of geriatric 3D was found as 10.6% (n = 44/415) for delirium, 45.6% (n = 160/351) for dementia, and 35.1% (n = 123/350) for depression. Delirium, dementia, and depression all had higher mortality rates among older ED patients covering the 5-year period. However, only delirium was predictive of both 6-month and 5-year mortality rates.

**Conclusion:**

Aside from the medical and surgical issues of geriatric patients, the prevalences of dementia and depression are much higher than expected in the emergency department. Delirium was a predictor for 6-month and 5-year mortality. We suggest that EDs should have screening tools for geriatric 3D mental health disorders to increase the quality of life for the geriatric population.

## 1. Introduction

The geriatric population in the world is growing fast, and the proportion of emergency department (ED) visits by older patients is expected to increase over the next decades [1]. ED is of importance due to becoming one of the first points of contact with many older patients, and mental health disorders are highly prevalent among older ED patients [2]. As a result, we can expect to see many patients with mental health disorders associated with the elderly, especially geriatric delirium, dementia, and depression (Geriatric 3D).

Geriatric 3D represents a common and challenging set of diagnoses for older patients. These disorders are interconnected, increasing the risk of each other, and are all associated with an increased risk of mortality and morbidity. Despite this, emergency physicians and other ED healthcare professionals do not routinely evaluate older patients for the disorders and may miss the critical diagnoses [3].

Several studies have addressed the prevalence and physician recognition of these mental health disorders in older patients presenting to the ED. However, to our knowledge, there was no study evaluating all three mental health disorders at the same time regarding the long-term survival after the ED admission. The aim of this study is to determine the prevalence of dementia, delirium, and depression among older patients presenting to the ED and to investigate the survival rate for 6-month (short-term) and 5-year (long-term) following the presentation to ED. In addition, we intend to draw attention to the silent epidemic in older ED patients.

## 2. Materials and methods

### 2.1. Study design and setting

This prospective observational cohort study was performed in an academic emergency department of a tertiary-care university hospital in the metropolitan city of Ankara, Turkey. Ankara is the capital city of Turkey, with a population of 5.2 million at the time of the study. Gazi University Hospital is one of the largest university hospitals with a capacity of 1100 hospital beds, including 97 intensive care unit beds. Our adult emergency department serves approximately 66,000 patients per year, with more than 12% older patients, stressing the capacity of ED [4]. The study was reviewed and approved by the Regional Clinical Research Ethics Committee of Education and Training Hospital within the scope of “Non-interventional Clinical Studies” (Number: 080715.930). The requirement for written informed consent was waived by the ethics committee, and verbal informed consent was obtained from all participants.

### 2.2. Study protocol

This study was conducted during a period of 30 days from December 10, 2014 to January 10, 2015 covering daytime (08:00–18:00) and night (18:00–08:00) ED shifts. The triage paramedic team notified the study team for all patients over the age of 65 presented to the ED via the hospital information technology system. The patients who presented during daytime on weekday shifts were interviewed in the study by the attending physicians I.K. and Z.U., and the patients who were presented during the weekday on night shifts and on weekend shifts were recruited in the study according to the shift scheduled by three trained interviewers: M.T., B.K., M.S.

#### 2.2.2. Inclusion and exclusion criteria of the study

**Figure 1:**
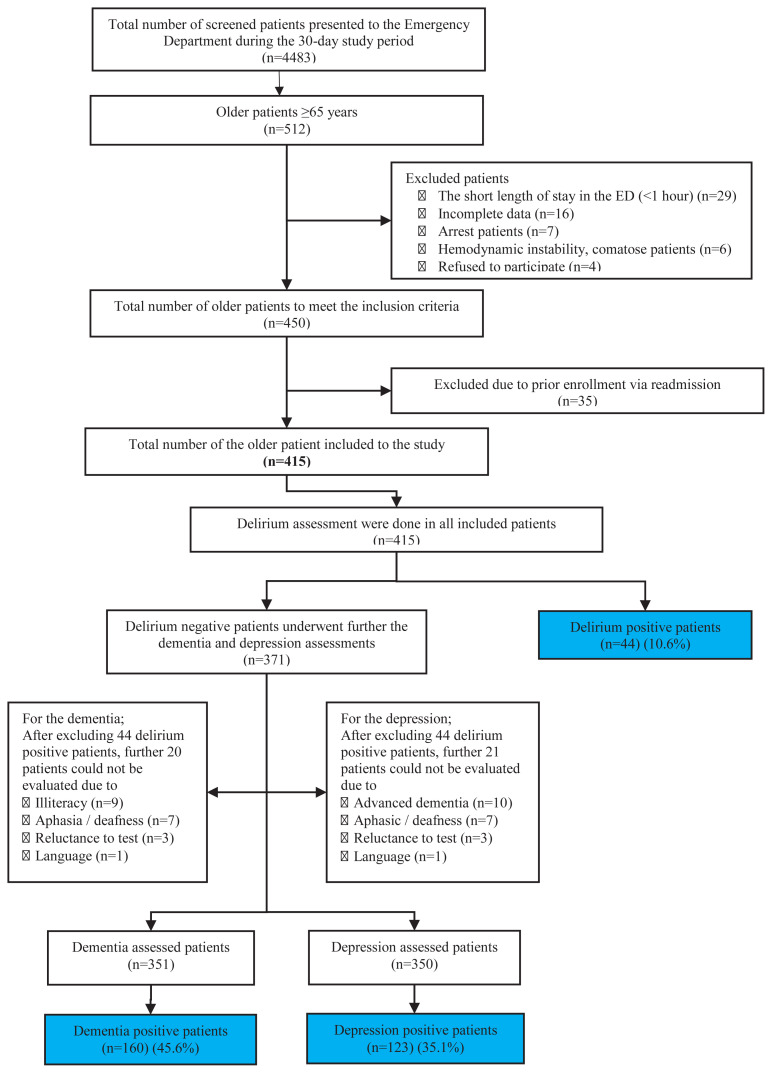
Patients’ flow chart.

All consecutive patients, aged 65 years or older, visiting the ED were recruited during the study period. Exclusion criteria were the patients who did not provide consent to participate, those who had a short length of stay (less than 1-hh) in the ED, those who were critically ill (hemodynamically unstable), those who presented with a cardiac arrest, or needed emergency resuscitation, and those with incomplete data acquisition during enrollment. Only the first visit was included for patients with repeated visits to the ED. The patient flow chart is described in Figure 1.

### 2.3. Data collection

#### 2.3.1. Patient characteristics

During the initial contact of the presentation in the ED, the demographics (age, sex, etc.), presenting complaints, admission by ambulance, accompanying relatives, comorbidities, medications, vital signs (body temperature, heart rate, systolic and diastolic blood pressures, oxygen saturation), and Glasgow Coma Score (GCS) were recorded in the patient data collection forms. Past medical history of diagnosed dementia and depression was also established at the time of the patient interview.

#### 2.3.2. Research interviewer training

Before commencing the study, all research interviewers were trained with a 4-h theoretical lecture to use geriatric mental disorder screening tools [delirium screening with Confusion Assessment Method (CAM), dementia screening with Quick Confusion Scale (QCS), and depression screening with Geriatric Depression Scale (GDS)] by the research coordinators. Subsequently, a pilot practical training session on mental health disorder tests was conducted with ten older patients for two consecutive days. Following training, all interviewers were supervised by an emergency medicine attending physician (I.K.) and a geriatric attending physician (Z.U.) for initial screens to ensure that the test was administered in a standardized manner. Any unclear queries were adjudicated by the attending physicians I. K. and Z.U. jointly.

#### 2.3.3. Geriatric 3D screening by trained interviewer

The screening of delirium, dementia, and depression of the patients were prospectively evaluated using the Confusion Assessment Method (CAM), Quick Confusion Scale (QCS), and short-form of Geriatric Depression Scale-15 (GDS), respectively. ED personnel were blinded to the findings of the assessment to avoid influencing treatment decisions.

Every patient was first assessed for delirium in the ED and Glasgow Coma Scale (GCS) was obtained. It was not possible to further assess dementia or depression for patients with acute delirium. If the patient does not have delirium and is engaging with the investigator, the evaluation was continued with dementia and depression screening tests. We should note that these screening tests have varying degree of sensitivities and specificities for exact diagnosis.

#### 2.3.4. Assessment of delirium

Delirium was assessed with the CAM developed by Inouye and colleagues. CAM is the most widely used delirium assessment tool in the ED with high sensitivity (94%–100%) and specificity (90%–95%) [5]. Monetta et al. have been widely referenced as a validation of the CAM [6]. The CAM consists of a four-item algorithm including (i) acute change in mental status, (ii) inattention, (iii) disorganized thinking, or (iv) altered level of consciousness [7]. Delirium diagnosis was made if a patient exhibits (i) and (ii) in the CAM, in addition to the presence of either (iii) or (iv). Since it is challenging to accurately diagnose delirium due to its fluctuating nature, relatives and family members were also interviewed to depict the detailed current and past medical history of the patients regarding the CAM characteristics.

#### 2.3.5. Assessment of dementia

The Quick Confusion Scale Assessment (QCS) was used to assess the level of cognitive functioning in the study. QCS is a six-item questionnaire to evaluate the orientation, memory, and concentration weighted to give a best total score of 15 [8]. Patients with a score of 11 or less were considered to have dementia. It has also been validated in the ED by Stair et al [9].

The presence of dementia was also confirmed by screening the past medical history, dementia medications and questioning the family and relatives. As previously described, QCS was inapplicable in delirious patients. If the patient did not have delirium, all study participants underwent the QCS test.

#### 2.3.6. Assessment of depression

Depression was assessed with the Short Form of Geriatric Depression Scale (GDS), specifically known as GDS-15. The GDS was originally 30-item questionnaires (GDS-30) created by Brink [10]. The GDS-15 was derived from the GDS-30, and it is designed to be used in older adults with short attention spans, who could get fatigued quickly. It consists of 15 questions derived from the validity and reliability study conducted by Yesavage et al [11]. It was also validated in Turkish older adult population by Ertan et al [12]. The time taken to perform the test is approximately 5–7 min [12]. A maximum of 15 points can be achieved. The cut-off score of 4/5 was accepted as depression positive in this study as recommended in the original validated study for interpreting the test results [13].

#### 2.3.7. Premorbid functional status assessment

Premorbid functional status was assessed by using the Barthel Index (BI). This widely used assessment is an ordinal scale measuring the ability of an individual to perform ten basic activities of daily living related to self-care, continence, and mobility, including bathing, grooming (0 and 5 points), feeding, dressing, fecal control, urinary control, climbing stairs (0, 5, and 10 points), transfers (i.e., from chair to bed), and walking (0, 5, 10, and 15 points) in an independent manner. The final score ranges from 0 (completely dependent) to 100 (totally independent) points with the 5-point intervals. BI scores were grouped further by functional categories using points; 0–20 total disability, 21–61 severe disability, 62–90 moderate disability, 91–99 mild disability, 100 points no disability.

#### 2.3.8. Assessment of preexisting comorbid conditions

The Charlson Comorbidity Index (CCI) was used to measure the comorbid burden. CCI, designed by Charlson and et al. in 1987, and it is a morbidity score that reflects mortality risk [14]. It is based on 19 different medical conditions categories. Each category was assigned to a score (weight) of 1,2,3, or 6. CCI was validated by Quan and et al [15].

#### 2.3.9. Clinical outcomes of the patients

The data for 6-month and 5-year mortality rates were collected from electronic medical records. Information on mortality regarding the date of death for patients was obtained from the Death Reporting System records (https://obs.saglik.gov.tr) developed by the Ministry of Health Information Technology, Turkey. Notification of deaths is obligatory in Turkey, and no burial can be carried out without this report approved by the State Physicians. The death report includes the information related to the identification numbers, identity information, date, and time of death of the individual.

### 2.4. Statistical analysis

The collected data were analyzed using SPSS software version 21 (IBM C, Chicago, IL, USA) and MedCalc version 15.8 (MedCalc Software bvba, Ostend, Belgium). Demographic and clinical variables were presented as descriptive statistics. The continuous variables were presented as median values, and interquartile ranges (IQRs), and the categorical variables were summarized as frequencies and percentages. The normality of the continuous variables was evaluated using the Kolmogorov–Smirnov test. The statistical differences between the two groups of continuous variables were determined using the Mann–Whitney U test. The categorical variables were compared using Pearson’s χ2 or Fisher’s exact test. The odds ratios (ORs) were presented with 95% confidence intervals (95% CIs). A critical α value of 0.05 was accepted as statistically significant.

Kaplan–Meier estimation was performed to generate the observed survival curves for geriatric 3D mental health disorders, and hazard ratios (HR) were calculated. Univariate and multivariate Cox regression analysis was applied to evaluate the prognostic relationship between mortality and geriatric 3D mental health disorders and other parameters. Only one of the parameters with a high correlation factor was included in this analysis.

## 3. Results

During the study, a total of 4463 patients presented to our ED. Of whom, 512 consecutive patients were at the age of 65 or older. 415 patients, meeting our inclusion criteria, were enrolled in the study (Figure 1). A total of 46.3% of the patients were male, median age was 74 years (IQR 68–80), and 21.2% presented to the ED with an ambulance. The most common comorbidities were hypertension (63.4%), diabetes mellitus (29.6%), coronary artery disease (25.5%), and malignancy (19%). In their past medical history, the rates of diagnoses of dementia and depression were 5.8% (n = 24) and 3.1% (n = 13), respectively. The 6-month mortality and 5-year mortality rates were 17.1% (n = 71) and 44.3% (n = 184) respectively. The geriatric assessment was completed within a median time of 20 minutes upon admission. The five most common admission complaints of the older patients are dyspnea (15.2%), abdominal pain (12.1%), and fall (10.9%), chest pain (8.1%), and fatigue (6.9%). Detailed demographic characteristics of the patients in the study is described in Table 1.

Among 415 patients included in the study, delirium evaluations were performed using CAM, and 44 (10.6%) delirium positivity was detected. Of these 415 patients, dementia assessment (QCS) could not be performed in 64 patients because 44 had acute delirium, 9 patients were illiterate, 7 patients had aphasia or deafness, 3 patients did not want to respond to dementia tests, and 1 patient had a language barrier. In the remaining 351 patients, dementia positivity was detected in 160 (45.6%) patients. In the same way, the depression test (GDS-15) could not be done in 65 patients because 44 patients had acute delirium, 10 patients had advanced dementia, 7 patients had aphasia or deafness, 3 patients did not want to respond to depression tests, and 1 patient had a language barrier. In the remaining 350 patients, depression positivity was detected in 123 (35.1%) patients.

### 3.1. Delirium

**Figure 2:**
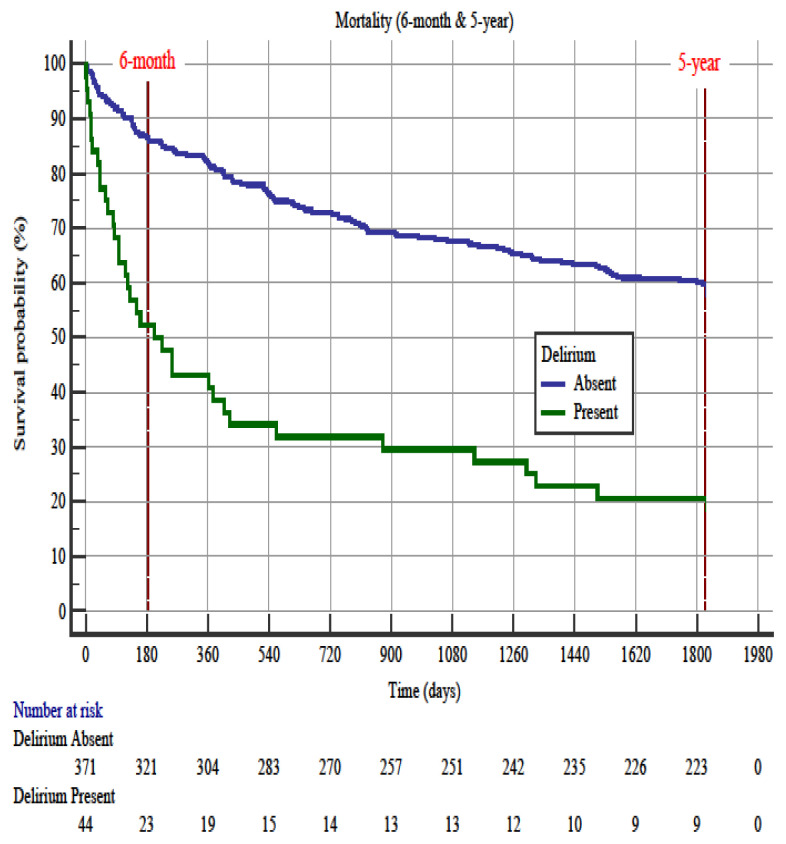
Kaplan–Meier survival curves in older emergency department patients with and without delirium for 6-month and 5-year. Hazard ratio of delirium on 6-month was 4.5 (95% CI 1.96–10.34) for 6-month mortality and was 3.4 (95% CI 1.90–6.22) for 5-year mortality.

The prevalence of delirium was 10.6% (n = 44/415). Hazard ratio of delirium was 4.5 (95% CI 1.96–10.34) for 6-month mortality and was 3.4 (95% CI 1.90–6.22) for 5-year mortality (Figure 2). In the Cox proportional hazards regression analysis, delirium (OR: 1.82, 95% CI 1.02–3.23), systolic blood pressure (OR: 0.99, 95% CI 0.98–0.99), and Barthel index group (OR: 0.59, 95% CI 0.49–0.72) were correlated with 6-month mortality. Following cox regression analysis for 5-year mortality, delirium (OR: 1.75, 95% CI 1.16–2.66), malignancy (OR: 1.76, 95% CI 1.11–2.78), Charlson comorbidity index (OR: 1.23, 95% CI 1.09–1.40), oxygen saturation (OR: 0.97, 95% CI 0.95–0.99), and Barthel index group (OR: 0.70, 95% CI 0.62–0.79) were the predictors (Table 2 and 3).

### 3.2. Dementia

**Figure 3:**
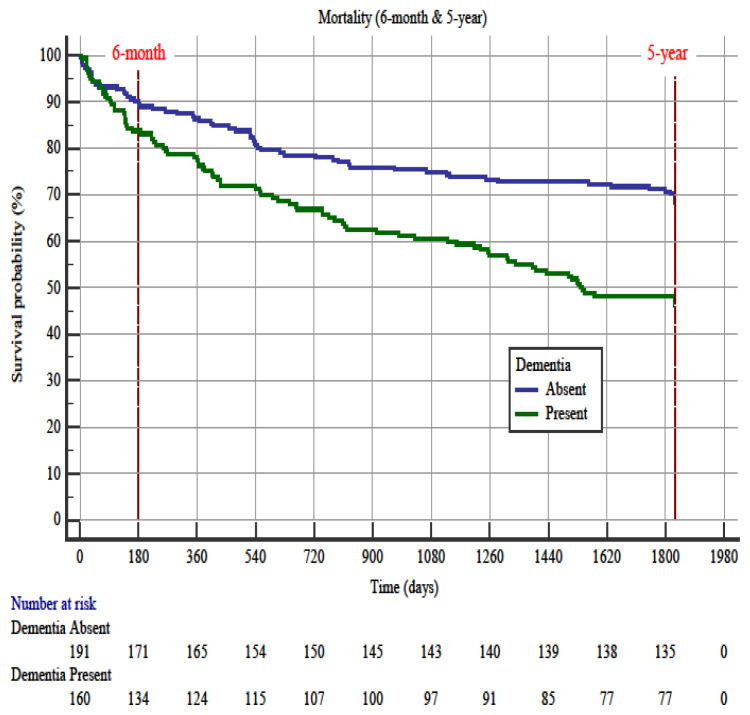
Kaplan–Meier survival curves in older emergency department patients with and without dementia for 6-month and 5-year. Hazard ratio of dementia was 1.6 (95% CI 0.89–2.84) for 6-month mortality and was 2.0 (95% CI 1.42–2.77) for 5-year mortality.

The prevalence for dementia was 45.6% (n = 160/351). The presence of dementia does not affect 6-month mortality in Kaplan–Meier survival analysis (OR: 1.6 with a 95% CI 0.89–2.84), (p = 0.1168). However, dementia positivity was relevant for the 5-year mortality (OR: 1.98 with a 95% CI 1.42–2.77) (p < 0.001) (Figure 3). In the Cox proportional hazards regression analysis, malignancy (OR: 3.00, 95% CI 1.45–6.18), coronary artery disease (OR: 2.16, 95% CI 1.11–4.16), and Barthel index group (OR: 0.68, 95% CI 0.53–0.86) were predictors for 6-month mortality. Following cox regression analysis for 5-year mortality, predictive factors were the malignancy (OR: 1.85, 95% CI 1.13–3.00), Charlson comorbidity index (OR: 1.16, 95% CI 1.02–1.32), oxygen saturation (OR: 0.97, 95% CI 0.94–0.99), and Barthel index group (OR: 0.71, 95% CI 0.63–0.86) (Table 4 and 5).

### 3.3. Depression

**Figure 4:**
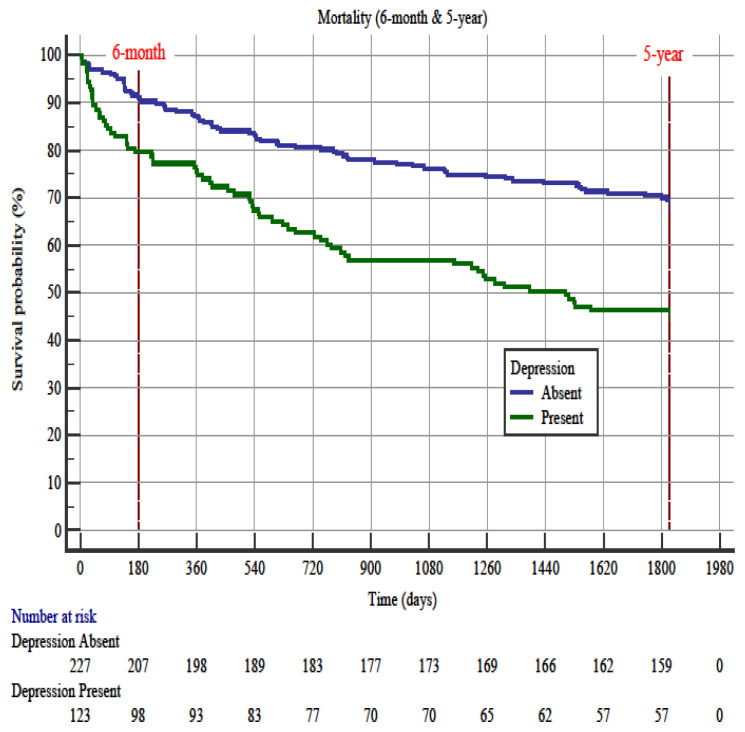
Kaplan-Meier survival curves in older emergency department patients with and without depression for 6-month and 5-year periods. The hazard ratio of depression was 2.5 (95% CI 1.34–4.64) for 6-month mortality and was 2.1 (95% CI 1.49–3.09) for 5-year mortality.

The prevalence of depression was 35.1% (n = 123/350). The hazard ratio of depression was 2.5 (95% CI 1.34–4.64) for 6-month mortality and 2.14 (95% CI 1.49–3.09) for 5-year mortality (Figure 4). In the Cox proportional hazards regression analysis for 6-month mortality, predictor factors were malignancy (OR: 3.33, 95% CI 1.62–6.81), coronary artery disease (OR: 2.09, 95% CI 1.08–4.03), and Barthel index group (OR: 0.69, 95% CI 0.54–0.87). Following Cox regression analysis for 5-year mortality, malignancy (OR: 1.97, 95% CI 1.19–3.24), Charlson comorbidity index (OR: 1.18, 95% CI 1.03–1.33), oxygen saturation (OR: 0.97, 95% CI 0.94–0.99), and Barthel index group (OR: 0.76, 95% CI 0.64–0.89) were the predictors (Table 6 and 7).

## 4. Discussion

In this study, we investigated the mental health disorders in the older patients presented to the ED, and we observed a silent epidemic with a prevalence of 10.6% for delirium, 45.6% for dementia, and 35.1% for depression, respectively. Considering that 3D mental health disorders are seen together with a high prevalence and have worse outcomes in terms of mortality, EDs have a vital role regarding screening of older adults after their presentation to the ED. EDs may be considered as the first point of contact for the screening of Geriatric 3D in many patients.

Older patients are the population at highest risk for decompensation if not diagnosed and managed early, due to reduced physiological reserve, atypical presentation of symptoms, more accompanying comorbidities, underlying fragility associated with aging, as well as hearing and visual impairments. Given the underlying medical fragility and complex presentation of older patients, the causes of mental and behavioral changes are likely to be misdiagnosed. In addition, they are likely to benefit from early intervention. For these reasons, our research questions have a significant potential impact on vulnerable older patients in the ED.

Delirium and agitation are among the most common problems in the geriatric population, occurring in approximately 25% of hospitalized geriatric patients [16, 17]. Patients with dementia are more likely to develop delirium [18], and patients with diagnosed delirium are more likely to develop dementia later in life [19]. Similarly, depression has been associated with an increased risk of developing subsequent dementia [20, 21]. Considering these three most common mental health disorders which are complex and multi-facets in older adults, our study provides information in terms of Geriatric 3D assessment and contributes to the limited data related to short-term and long-term survival rates.

Delirium in the ED is widely studied in the literature. According to reported studies, the delirium prevalence in patients older than 65 years of age presenting to ED is reported as 8.3% [22] and 12% [23]. In a systematic review done by Barron, the prevalence of delirium at admission to the ED was reported as 7% to 20% [24]. Our finding of 10.6% for delirium prevalence is in accordance with the literature. We would like to point out that we used CAM for depression screening in our older ED patients. The CAM is the most widely used standardized and validated method for the identification of delirium with high sensitivity and specificity [5].

In our study, the prevalence of dementia was found to be relatively high. In the literature, the prevalence of dementia in EDs was reported to be between 20% and 38% [25]. There may be several reasons for the high prevalence of dementia screening. First, our emergency department, where our study was conducted, has a larger older adult population, and the density of older patients coming from nursing homes can be a contributing factor. Another reason may be “The Quick Confusion Scale” instrument, which we used to screen for dementia. At the beginning of the study, we planned to use the Mini-Mental State test (MMSE) for dementia screening. However, the application of this test was not very successful due to the lower education level of most of our older patients. The accompanying fatigue and general illness in many of the older patients makes it difficult to complete the “clock drawing” phase of the MMSE components. Alternatively, several brief methods of assessing global cognition have been evaluated in the ED setting like Alzheimer Disease-8 (caregiver completed) [26], Ottawa 3DY [27], 6-item Screener [28], Short Blessed Test [26], QCS [8], Mini-Cog [29]. The use of QCS has been validated in an independent emergency department by Stair et al [9]. Furthermore, it is easier to administer the QCS because it does not have any prerequisite for reading, writing, or drawing. Nevertheless, based on the criteria of the fifth edition of the “Diagnostic and Statistical Manual of Mental Disorders” in dementia diagnosis, there is no clear screening test with proven clinical efficacy. Therefore, we preferred to use the QCS test for dementia screening [9].

Depression in older patients, another important topic of our study, is common and untreated in this population. It may coexist with dementia and increases the risk of delirium. It is also associated with higher morbidity and mortality rates, together with increased healthcare utilization. Depression may be present in up to one-third of older ED patients [30, 31]. In our study, we used the GDS-15 questionnaire for the screening of geriatric depression in the patients. GDS-15 is one of the most widely used and reliable scales for the assessment of depression in older patients. The GDS-15 was first created in response to the need for a diagnostic instrument, specifically aimed at the elderly, which could distinguish a patient suffering from the depression from other mental health disorders such as cognitive impairment [32, 33]. Besides GDS, there are several screening tests for depression currently available including the Patient Health Questionnaire-2 [34], Patient Health Questionnaire-9 Self-Assessment Tool [35], Beck Depression Inventory for Primary Care [36]. The two simple questions in the Patient Health Questionnaire-2 have been validated as a screening tool for depressive disorders in the primary care setting [34], but may also be useful in the ED.

Our finding of depression prevalence is 35.1%. It is higher when compared to the studies done in the ED of developed countries [16.5% (USA) [37], 17% (USA) [38], 22.8% (Australia) [39], 27% (USA) [30], 32% (USA) [40]]. However, in a recent study carried out in Nepal, one of the low-income countries, the prevalence of depression in geriatric patients presenting to ED was reported as 45.7% [32]. The differences in the prevalence of depression may also be affected by being low, middle, or high-income countries, as well as different screening tools used.

Another aim of this study was to investigate whether geriatric 3D has an effect on short-term (6-month) and long-term (5-year) mortality. In our study, we found that geriatric patients diagnosed with one of the mental health disorders had higher mortality rates than those without, and delirium alone predicted mortality in both 6-month and 5-year periods. In literature, Kakuma and et al. reported that inability to detect delirium in the ED might be associated with increased mortality within 6 months after discharge [41]. Also, Han and et al. reported that the diagnosed delirium in the ED was an independent factor of increased 6-month mortality rate (HR=1.72 with 95% CI 1.04–2.86) [42]. Similarly, in our study, the presence of delirium had a predictive role for both 6-month (HR:4.5, 95% CI 1.96–10.34) and 5-year mortality (HR:3.4, 95% CI 1.90–6.22).

Older adults with dementia have a higher rate of ED admission, hospitalization, and ED revisits [43]. A large study database of older patients, published by LaMantia et al. from China, reported that older adults with dementia have higher mortality after an ED visit than patients without dementia. They also stated that the survival and ED return differed according to the dementia status of the patient [44]. In our study, dementia positivity was correlated with the 5-year mortality; however, no difference was found in the univariate analysis in terms of 6-month mortality. It suggests that dementia is a chronic disease associated with long-term mortality.

The relationship between the diagnosis of depression and mortality in the ED was also examined, and mortality was found to be high in patients diagnosed with depression [44]. In our study, patients with depression had a higher mortality rate at 6-month and 5-year. However, in multivariate Cox proportional hazard ratio testing for 6-month and 5-year mortality, especially malignancy existence, premorbid functional dependence, and co-morbidities were predictors for the mortality. This may be due to the fact that depression is seen together with other co-existing underlying diseases, rather than having a direct mortality effect.

### 4.1. Study limitations

Our findings have to be considered in conjunction with the study limitations. First, we used three validated instruments to identify the geriatric 3D, the CAM for delirium diagnosis, QCS for dementia, GDS for depression as a reference standard in our study. While they are widely used for research and clinical purposes, they are not “gold standard” diagnostic instruments. Thus, our results should be interpreted with caution by the possibility of an imperfect gold standard bias [45]. Secondly, our study was performed at a single study site, which is a tertiary referral academic center, and our findings may not be applied to generalized rural or non-academic EDs. Moreover, the diagnostic bias might have skewed the estimated prevalence. Lastly, some of the geriatric 3D assessments were performed by the junior research doctors instead of the geriatrician. However, we applied strict documentation methods, checked every document thoroughly, and assessed all patients with the help of senior geriatric and emergency medicine attending physicians.

## 5. Conclusion

Apart from medical and surgical problems of the geriatric patient population, incidences of dementia and depression are much higher than expected in the emergency department. We suggest that emergency departments should have screening tools for dementia, delirium, and depression (3D) to increase the quality of life for the geriatric patient population.

## Figures and Tables

**Table 1:** The characteristics of older emergency department patients included to the study according to the delirium, dementia, and depression positive group (n = 415).

	Total number of patients (n = 415)	Delirium positive (n = 44)	Dementia positive (n = 160)	Depression positive (n = 123)
**Age, years (IQR 25–75)**	74 (68–80)	81 (73.3–86.0)	76 (71–84)	74 (68–78)
**Sex, n (%)**				
·Male	192 (46.3)	20 (45.5)	61 (38.1)	55 (44.7)
**Admission by ambulance, n (%)**	88 (21.2)	24 (54.5)	35 (21.9)	25 (20.3)
**Comorbidities, n (%)**				
·Hypertension	263 (63.4)	28 (63.6)	108 (67.5)	75 (61.0)
·Diabetes Mellitus	123 (29.6)	14 (31.8)	47 (29.4)	38 (30.9)
·Coronary Artery Disease	106 (25.5)	11 (25.0)	53 (33.1)	38 (30.9)
·Malignancy	79 (19.0)	11 (25.0)	29 (18.1)	36 (29.3)
·Chronic Obstructive Pulmonary Disease	51 (12.3)	10 (22.7)	20 (12.5)	23 (18.7)
·Congestive Heart Failure	49 (11.8)	14 (31.8)	19 (11.9)	15 (12.2)
·Cerebrovascular Disease	34 (8.2)	5 (11.4)	20 (12.5)	10 (8.1)
·Atrial Fibrillation	33 (8.0)	4 (9.1)	19 (11.9)	14 (11.4)
·Asthma	29 (7.0)	0 (0)	17 (10.6)	8 (6.5)
·Prostate Hypertrophy	28 (6.7)	4 (9.1)	7 (4.4)	5 (4.1)
·Hypothyroidism	28 (6.7)	5 (11.4)	13 (8.1)	7 (5.7)
·Chronic Renal Failure	23 (5.5)	4 (9.1)	8 (5.0)	7 (5.7)
·Cholelithiasis	18 (4.3)	0 (0)	9 (5.6)	8 (6.5)
·Long Term Oxygen Therapy	12 (2.9)	4 (9.1)	5 (3.1)	5 (4.1)
·Parkinson	11 (2.7)	4 (9.1)	6 (3.8)	5 (4.1)
·Chronic Liver Disease	4 (1.0)	1 (2.3)	1 (0.6)	0 (0)
**History of Mental Health Disorders, n (%)**				
·Dementia in the Past Medical History	24 (5.8)	8 (18.2)	15 (9.4)	3 (2.4)
·Depression in the Past Medical History	13 (3.1)	3 (6.8)	3 (1.9)	10 (8.1)
**Vital Signs at the time of ED Admission**				
·Body temperature (°C) (IQR 25–75)	36.4 (36.0–36.6)	36.5 (36.2–36.6)	36.4 (36–36.6)	36.4 (36–36.5)
·Pulse (/min) (IQR 25–75)	83 (74–96)	84.5 (67–96)	82.5 (74–96)	86 (76–99)
·Systolic pressure (mmHg) (IQR 25–75)	140 (120–160)	120 (102.5–149)	140 (120–160)	136 (120–160)
·Diastolic pressure (mmHg) (IQR 25–75)	80 (70–90)	70 (60–80)	80 (70–90)	80 (70–90)
·Oxygen saturation (%) (IQR 25–75)	96 (93–97)	93.5 (86–96.8)	95 (93–97)	95 (92–97)
·Glasgow coma score, (IQR 25–75)	15 (15–15)	13 (12–15)	15 (15–15)	15 (15–15)
**Barthel index**	100 (65–100)	60 (13–89)	90 (51–100)	90 (50–100)
·Barthel index Grouped	5 (3–5)	2.5 (1–3.75)	4 (2–5)	4 (2–5)
**Charlson comorbidity index**	5 (3–6)	6 (5–8)	5 (4–6)	5 (4–6)
**Disposition, n (%)**				
·Discharged	172 (41.4)	4 (9.1)	62 (38.8)	46 (37.4)
·Admitted to the hospital	292 (48.7)	29 (65.9)	80 (50.0)	61 (49.6)
·Admitted to Intensive Care Unit	38 (9.2)	11 (25)	17 (10.6)	15 (12.2)
·Voluntarily discharged	3 (0.7)	0 (0)	1 (0.6)	1 (0.8)
**Mortality, n (%)**				
·6–month mortality	71 (17.1)	**21 (47.7)**	26 (16.3)	25 (20.3)
·5-year mortality	184 (44.3)	**35 (79.5)**	83 (51.9)	66 (53.7)

**Table 2:** Univariate and cox proportional hazard regression analysis of older delirium patients regarding 6-month mortality.

	6-month mortality (−)	6-month mortality (+)	p	Odds Ratio	95% CI for Odds ratio	P
**Age, years (IQR 25–75)**	73.5 (68–80)	76 (70–84)	0,023	0.98	0.95–1.02	0.348
**Male, n (%)**	157 (45.6)	35 (49.3)	0,574			
**Delirium, n (%)**	23 (6.7)	21 (29.6)	<0.001	**1.82**	**1.02–3.23**	**0.043**
**Comorbidities, n (%)**						
·Hypertension	220 (64.0)	43 (60.6)	0.589			
·Diabetes Mellitus	99 (28.8)	24 (33.8)	0.399			
·Coronary Artery Disease	82 (23.8)	24 (33.8)	0.80			
·Malignancy	51 (14.8)	28 (39.4)	<0.001	1.74	0.95–3.20	0.076
·Chronic Obstructive Pulmonary Disease^*^	37 (10.8)	14 (19.7)	0.036	1.07	0.48–2.41	0.855
·Congestive Heart Failure	33 (9.6)	16 (22.5)	0.002	1.00	0.52–1.94	0.993
·Cerebrovascular Disease	27 (7.8)	7 (9.9)	0.574			
·Atrial Fibrillation	26 (7.6)	7 (9.9)	0.514			
·Asthma	28 (8.1)	1 (1.4)	0.041	0.26	0.04–1.97	0.196
·Prostate Hypertrophy	22 (6.4)	6 (8.5)	0.602			
·Hypothyroidism	22 (6.4)	6 (8.5)	0.602			
·Chronic Renal Failure	18 (5.2)	5 (7.0)	0.568			
·Cholelithiasis	14 (4.1)	4 (5.6)	0.526			
·Long Term Oxygen Therapy	7 (2)	5 (7)	0.038	0.74	0.24–2.26	0.59
·Parkinson	9 (2.6)	2 (2.8)	1.000			
·Chronic Liver Disease	2 (0.6)	2 (2.8)	0.137			
**History of Mental Health Disorders, n (%)**						
·Dementia in the Past Medical History	16 (4.7)	8 (11.3)	0.046	0.72	0.32–1.64	0.44
·Depression in the Past Medical History	10 (2.9)	3 (4.2)	0.473			
**Vital Signs at the time of ED Admission**						
·Body temperature (°C) (IQR 25–75)	36.4 (36.0–36.6)	36.4 (36.2–36.6)	0,978			
·Pulse (/min) (IQR 25–75)	82 (74–96)	86 (82–90)	0.391			
·Systolic pressure (mmHg) (IQR 25–75)	140 (120–160)	121 (110–147)	<0.001	**0.99**	**0.98–0.99**	**0.034**
·Diastolic pressure (mmHg) (IQR 25–75)	80 (70–90)	80 (60–90)	0.224			
·Oxygen saturation (%) (IQR 25–75)	96 (94–98)	94 (86.5–96.8)	<0.001	0.97	0.94–1.00	0.065
·Glasgow coma score, (IQR 25–75)	15 (15–15)	15 (15–15)	<0.001			
**Barthel Index Group**	5 (3–5)	2 (2–4)	<0.001	**0.59**	**0.49–0.72**	**<0.001**
**Charlson comorbidity index**	4 (3–5)	6 (5–8)	<0.001	1.14	0.99–1.30	0.071

**Table 3:** Univariate and cox proportional hazard regression analysis of older delirium patients regarding 5-year mortality.

	5-year mortality (−)	5-year mortality (+)	p	Odds Ratio	95% CI for Odds ratio	P
**Age, years (IQR 25–75)**	72 (67–76)	78 (70–84)	<0.001	1.02	1.00–1.05	0.052
**Male, n (%)**	99 (42.9)	93 (50.5)	0.119			
**Delirium, n (%)**	9 (3.9)	35 (19.0)	<0.001	**1.75**	**1.16–2.66**	**0.008**
**Comorbidities, n (%)**						
·Hypertension	154 (66.7)	109 (59.2)	0.119			
·Diabetes Mellitus	60 (26.0)	63 (34.2)	0.067	0.93	0.64–1.35	0.690
·Coronary Artery Disease	57 (24.7)	49 (26.6)	0.650			
·Malignancy	26 (11.3)	53 (28.8)	<0.001	**1.76**	**1.11–2.78**	**0.017**
·Chronic Obstructive Pulmonary Disease	18 (7.8)	33 (17.9)	0.002	1.13	0.68–1.86	0.636
·Congestive Heart Failure	10 (4.3)	39 (21.2)	<0.001	1.09	0.71–1.66	0.705
·Cerebrovascular Disease	14 (6.1)	20 (10.9)	0.076	0.70	0.40–1.25	0.239
·Atrial Fibrillation	14 (6.1)	19 (10.3)	0.111			
·Asthma	19 (8.2)	10 (5.4)	0.268			
·Prostate Hypertrophy	13 (5.6)	15 (8.2)	0.308			
·Hypothyroidism	15 (6.5)	13 (7.1)	0.818			
·Chronic Renal Failure	7 (3.0)	16 (8.7)	0.012	0.82	0.44–1.52	0.526
·Cholelithiasis	11 (4.8)	7 (3.8)	0.634			
·Long Term Oxygen Therapy	1 (0.4)	11 (6.0)	0.001	1.25	0.58–2.68	0.572
·Parkinson	2 (0.9)	9 (4.9)	0.014	1.24	0.62–2.49	0.551
·Chronic Liver Disease	1 (0.4)	3 (1.6)	0.327			
**History of Mental Health Disorders, n (%)**						
·Dementia in the Past Medical History	4 (1.7)	20 (10.9)	<0.001	0.90	0.51–1.60	0.727
·Depression in the Past Medical History	8 (3.5)	5 (2.7)	0.665			
**Vital Signs at the time of ED Admission**						
·Body temperature (°C) (IQR 25–75)	36.4 (36.0–36.6)	36.4 (36.0–36.6)	0,424			
·Pulse (/min) (IQR 25–75)	81 (74–95)	84 (74–97)	0,443			
·Systolic pressure (mmHg) (IQR 25–75)	140 (120–160)	131 (113–160)	0,010	1.00	0.99–1.00	0.291
·Diastolic pressure (mmHg) (IQR 25–75)	80 (70–90)	80 (65–90)	0.025			
·Oxygen saturation (%) (IQR 25–75)	96 (95–98)	95 (91–97)	<0.001	**0.97**	**0.95–0.99**	**0.008**
·Glasgow coma score, (IQR 25–75)	15 (15–15)	15 (15–15)	<0.001			
**Barthel Index Group**	5 (5–5)	3 (2–5)	<0.001	**0.70**	**0.62–0.79**	**0.001**
**Charlson comorbidity index**	4 (3–5)	5 (4–7)	<0.001	**1.23**	**1.09–1.40**	**0.001**

**Table 4:** Univariate and cox proportional hazard regression analysis of older dementia patients regarding 6-month mortality.

	6-month mortality (−)	6-month mortality (+)	p	Odds Ratio	95% CI for Odds ratio	P
**Age, years (IQR 25–75)**	73 (68–79)	74.5 (69–79)	0.379			
**Male, n (%)**	142 (46.6)	25 (54.3)	0.324			
**Dementia, n (%)**	134 (43.9)	26 (56.5)	0.110			
**Comorbidities, n (%)**						
·Hypertension	193 (63.3)	27 (58.7)	0.549			
·Diabetes Mellitus	85 (27.9)	16 (34.8)	0.334			
·Coronary Artery Disease	73 (23.9)	19 (41.3)	0.013	**2.16**	**1.11–4.16**	**0.023**
·Malignancy	47 (15.4)	19 (41.3)	<0.001	**3.00**	**1.45–6.18**	**0.003**
·Chronic Obstructive Pulmonary Disease^*^	34 (11.1)	5 (10.9)	0.955			
·Congestive Heart Failure	27 (8.9)	8 (17.4)	0.072	1.07	0.47–2.41	0.864
·Cerebrovascular Disease	23 (7.5)	4 (8.7)	0.784			
·Atrial Fibrillation	24 (7.9)	5 (10.9)	0.491			
·Asthma	28 (9.2)	1 (2.2)	0.108			
·Prostate Disease	19 (6.2)	4 (8.7)	0.522			
·Hypothyroidism	21 (6.9)	2 (4.3)	0.752			
·Chronic Renal Failure	15 (4.9)	4 (8.7)	0.292			
·Cholelithiasis	12 (3.9)	4 (8.7)	0.143			
·Long Term Oxygen Therapy	7 (2.3)	1 (2.2)	1.000			
·Parkinson	5 (1.6)	2 (4.3)	0.230			
·Chronic Liver Disease	2 (0.7)	0 (0.0)	1.000			
**History of Mental Health Disorders, n (%)**						
·Dementia in the Past Medical History	10 (3.3)	5 (10.9)	0.034	1.69	0.60–4.71	0.316
·Depression in the Past Medical History	7 (2.3)	0 (0.0)	0.601			
**Vital Signs at the time of ED Admission**						
·Body temperature (°C) (IQR 25–75)	36.4 (36–36.6)	36.4 (36–36.7)	0.818			
·Pulse (/min) (IQR 25–75)	82 (74–96)	84 (76–96)	0.868			
·Systolic pressure (mmHg) (IQR 25–75)	140 (120–160)	130 (119–147)	0.015	0.99	0.97–1.00	0.130
·Diastolic pressure (mmHg) (IQR 25–75)	80 (70–90)	80 (67–90)	0.585			
·Oxygen saturation (%) (IQR 25–75)	96 (94–98)	945 (92–96)	0.029	0.98	0.94–1.02	0.423
·Glasgow coma score, (IQR 25–75)	15 (15–15)	15 (15–15)	0.783			
**Barthel Index Group**	5 (3–5)	3 (2–5)	<0.001	**0.68**	**0.53–0.86**	**0.001**
**Charlson comorbidity index**	4 (3–5)	5 (5–7)	<0.001	1.03	0.86–1.23	0.704

**Table 5:** Univariate and cox proportional hazard regression analysis of older dementia patients regarding 5-year mortality.

	5-year mortality (−)	5-year mortality (+)	p	Odds Ratio	95% CI for Odds ratio	P
**Age, years (IQR 25–75)**	72 (67–76)	76 (70–84)	<0.001	1.02	0.99–1.05	0.151
**Male, n (%)**	94 (44.5)	73 (52.1)	0.163			
**Dementia, n (%)**	77 (36.5)	83 (59.3)	<0.001	1.05	0.71–1.56	0.796
**Comorbidities, n (%)**						
·Hypertension	138 (65.4)	82 (58.6)	0.195			
·Diabetes Mellitus	51 (24.2)	50 (35.7)	0.019	1.27	0.83–1.92	0.268
·Coronary Artery Disease	50 (23.7)	41 (30.0)	0.189			
·Malignancy	26 (12.3)	40 (28.6)	<0.001	**1.85**	**1.13–3.00**	**0.015**
·Chronic Obstructive Pulmonary Disease^*^	17 (8.1)	22 (15.7)	0.025	1.00	0.57–1.74	0.991
·Congestive Heart Failure	8 (3.8)	27 (19.3)	<0.001	1.41	0.86–2.29	0.175
·Cerebrovascular Disease	13 (6.2)	14 (10.0)	0.186			
·Atrial Fibrillation	13 (6.2)	16 (11.4)	0.079	0.95	0.51–1.72	0.855
·Asthma	19 (9.0)	10 (7.1)	0.535			
·Prostate Disease	13 (6.2)	10 (7.1)	0.716			
·Hypothyroidism	15 (7.1)	8 (5.7)	0.605			
·Chronic Renal Failure	7 (3.3)	12 (8.6)	0.033	1.09	0.53–2.19	0.817
·Cholelithiasis	9 (4.3)	7 (5.0)	0.747			
·Long Term Oxygen Therapy	1 (0.5)	7 (5.0)	0.005	1.05	0.41–2.67	0.919
·Parkinson	1 (0.5)	6 (4.3)	0.012	1.24	0.52–2.93	0.626
·Chronic Liver Disease	1 (0.5)	1 (0.7)	0.770			
**History of Mental Health Disorders, n (%)**						
·Dementia in the Past Medical History	3 (1.4)	12 (8.6)	<0.001	1.22	0.60–2.46	0.589
·Depression in the Past Medical History	6 (2.8)	1 (0.8)	0.162			
**Vital Signs at the time of ED Admission**						
·Body temperature (°C) (IQR 25–75)	36.4 (36–36.6)	36.3 (36–36.6)	0.217			
·Pulse (/min) (IQR 25–75)	82 (75–95)	84 (74–97)	0.920			
·Systolic pressure (mmHg) (IQR 25–75)	140 (120–160)	130 (119–147)	0.015	1.00	0.98–1.00	0.134
·Diastolic pressure (mmHg) (IQR 25–75)	80 (70–90)	80 (68–90)	0.033			
·Oxygen saturation (%) (IQR 25–75)	96 (95–98)	95 (91–97)	<0.001	**0.97**	**0.94–0.99**	**0.008**
·Glasgow coma score, (IQR 25–75)	15 (15–15)	15 (15–15)	0.385			
**Barthel Index Group**	5 (5–5)	3 (2–5)	<0.001	**0.71**	**0.63–0.86**	**<0.001**
**Charlson comorbidity index**	4 (3–5)	5 (4–7)	<0.001	**1.16**	**1.02–1.32**	**0.021**

**Table 6:** Univariate and cox proportional hazard regression analysis of older study patients with depression regarding 6-month mortality.

	6-month mortality (−)	6-month mortality (+)	p	Odds Ratio	95% CI for Odds ratio	P
**Age, years (IQR 25–75)**	73 (68–78)	74 (69–79)	0.609			
**Male, n (%)**	140 (45.9)	23 (51.1)	0.513			
**Depression, n (%)**	98 (32.1)	25 (55.6)	0.002	1.33	0.70–2.50	0.385
**Comorbidities, n (%)**						
·Hypertension	197 (64.6)	28 (62.2)	0.757			
·Diabetes Mellitus	87 (28.5)	16 (35.6)	0.334			
·Coronary Artery Disease	76 (24.9)	19 (42.2)	0.015	**2.09**	**1.08–4.03**	**0.029**
·Malignancy	44 (14.4)	21 (46.7)	<0.001	**3.33**	**1.62–6.81**	**0.001**
·Chronic Obstructive Pulmonary Disease^*^	33 (10.8)	6 (13.3)	0.617			
·Congestive Heart Failure	26 (8.5)	8 (17.8)	0.060	1.16	0.51–2.63	0.719
·Cerebrovascular Disease	21 (6.9)	3 (6.7)	1.000			
·Atrial Fibrillation	23 (7.5)	5 (111)	0.382			
·Asthma	27 (8.9)	1 (2.2)	0.151			
·Prostate Hypertrophy	19 (6.2)	4 (8.9)	0.516			
·Hypothyroidism	21 (6.9)	2 (4.4)	0.752			
·Chronic Renal Failure	14 (4.6)	4 (8.9)	0.267			
·Cholelithiasis	14 (4.6)	4 (8.9)	0.267			
·Long Term Oxygen Therapy	6 (2.0)	1 (2.2)	1.000			
·Parkinson	5 (1.6)	2 (4.4)	0.223			
·Chronic Liver Disease	2 (0.7)	0 (0.0)	1.000			
**History of Mental Health Disorders, n (%)**						
·Dementia in the Past Medical History	8 (2.6)	3 (6.7)	0.156			
·Depression in the Past Medical History	9 (3.0)	1 (2.2)	1.000			
**Vital Signs at the time of ED Admission**						
·Body temperature (°C) (IQR 25–75)	36.4 (36–36.6)	36.4 (36–36.7)	0.657			
·Pulse (/min) (IQR 25–75)	82 (74–96)	84 (76–97)	0.633			
·Systolic pressure (mmHg) (IQR 25–75)	140 (120–160)	130 (113–147)	0.005	0.99	0.97–1.00	0.104
·Diastolic pressure (mmHg) (IQR 25–75)	80 (70–90)	80 (64–90)	0.261			
·Oxygen saturation (%) (IQR 25–75)	96 (94–98)	95 (92–97)	0.040	0.99	0.95–1.03	0.677
·Glasgow coma score, (IQR 25–75)	15 (15–15)	15 (15–15)	1.000			
**Barthel Index Group**	5 (3–5)	3 (2–5)	<0.001	**0.69**	**0.54–0.87**	**0.002**
**Charlson comorbidity index**	4 (3–5)	5 (5–7)	<0.001	1.05	0.88–1.24	0.586

**Table 7:** Univariate and Cox proportional hazard regression analysis of older study patients with depression regarding 5-year mortality.

	5-year mortality (−)	5-year mortality (+)	p	Odds Ratio	95% CI for Odds ratio	P
**Age, years (IQR 25–75)**	72 (67–76)	76 (70–83)	<0.001	1.02	0.99–1.05	0.137
**Male, n (%)**	95 (44.2)	70 (50.4)	0.259			
**Depression, n (%)**	57 (26.5)	66 (48.9)	<0.001	1.18	0.81–1.74	0.399
**Comorbidities, n (%)**						
·Hypertension	143 (66.5)	82 (60.7)	0.273			
·Diabetes Mellitus	54 (25.1)	49 (36.3)	0.025	1.25	0.81–1.89	0.307
·Coronary Artery Disease	53 (24.7)	42 (31.1)	0.186			
·Malignancy	24 (11.2)	41 (30.4)	<0.001	**1.97**	**1.19–3.24**	**0.009**
·Chronic Obstructive Pulmonary Disease^*^	18 (8.4)	21 (15.6)	0.038	0.95	0.54–1.67	0.873
·Congestive Heart Failure	8 (3.7)	26 (19.3)	<0.001	1.44	0.87–2.36	0.153
·Cerebrovascular Disease	12 (5.6)	12 (8.9)	0.233			
·Atrial Fibrillation	13 (6.0)	15 (11.1)	0.089	0.99	0.52–1.84	0.966
·Asthma	18 (8.4)	10 (7.4)	0.746			
·Prostate Hypertrophy	13 (6.0)	10 (7.4)	0.617			
·Hypothyroidism	15 (7.0)	8 (5.9)	0.699			
·Chronic Renal Failure	7 (3.3)	11 (8.1)	0.044	1.07	0.51–2.21	0.856
·Cholelithiasis	11 (5.1)	7 (5.2)	0.977			
·Long Term Oxygen Therapy	1 (0.5)	6 (4.4)	0.015	1.04	0.38–2.79	0.934
·Parkinson	1 (0.5)	6 (4.4)	0.015	1.33	0.55–3.15	0.524
·Chronic Liver Disease	1 (0.5)	1 (0.7)	1.000			
**History of Mental Health Disorders, n (%)**						
·Dementia in the Past Medical History	3 (1.4)	8 (5.9)	0.018	1.06	0.45–2.44	0.900
·Depression in the Past Medical History	8 (3.7)	2 (1.5)	0.328			
**Vital Signs at the time of ED Admission**						
·Body temperature (°C) (IQR 25–75)	36.4 (36–36.6)	36.4 (36–36.6)	0.425			
·Pulse (/min) (IQR 25–75)	82 (75–96)	84 (74–96)	0.994			
·Systolic pressure (mmHg) (IQR 25–75)	140 (120–160)	139 (113–160)	0.088	1.00	0.98–1.00	0.153
·Diastolic pressure (mmHg) (IQR 25–75)	80 (70–90)	80 (65–90)	0.020			
·Oxygen saturation (%) (IQR 25–75)	96 (95–98)	95 (92–97)	<0.001	**0.97**	**0.94–0.99**	**0.012**
·Glasgow coma score, (IQR 25–75)	15 (15–15)	15 (15–15)	1.000			
**Barthel Index Group**	5 (5–5)	3 (2–5)	<0.001	**0.76**	**0.64–0.89**	**0.001**
**Charlson comorbidity index**	4 (3–5)	5 (4–7)	<0.001	**1.18**	**1.03–1.33**	**0.016**
